# Supplementary material for: Typhoid toxin exhausts the RPA response to DNA replication stress driving senescence and Salmonella infection
Source: Nat Commun. 2019 Sep 6;10:4040. doi: 10.1038/s41467-019-12064-1 (PMC6731267; doi:10.1038/s41467-019-12064-1)
Supplement: Supplementary file 1 — Supplementary Information [file 41467_2019_12064_MOESM1_ESM.pdf]

Supplementary Information

Typhoid toxin exhausts the RPA response to DNA replication stress driving senescence and *Salmonella* infection

Ibler et al

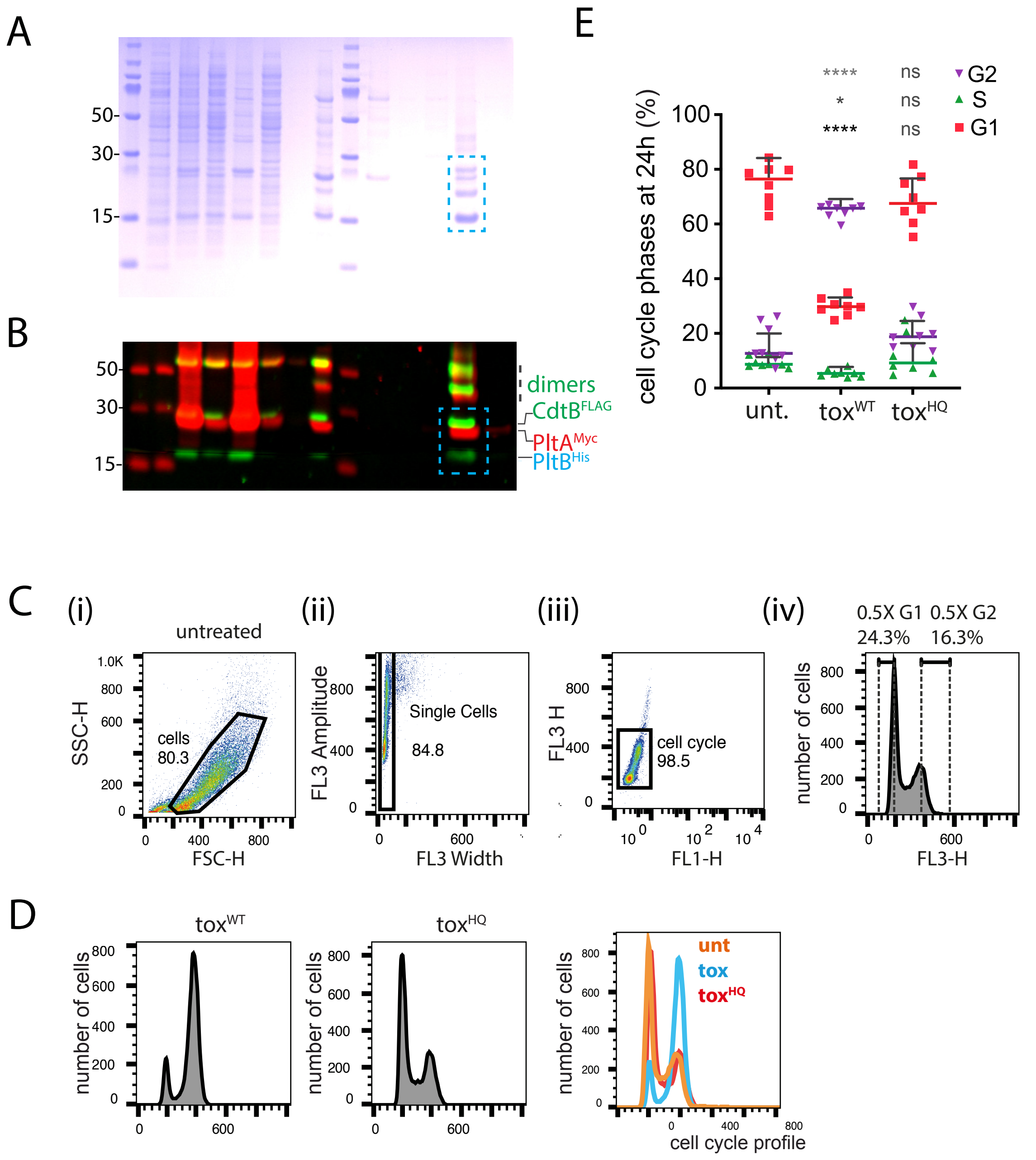

Supplementary Figure 1. Recombinant typhoid toxin induces cell-cycle arrest.

Unprocessed SDS-PAGE gel (**A**) and immunoblot (**B**) used to generate Fig 1A and 1B. Crop indicated with blue box. CdtB and PltA dimers indicated in B. MW in kDa left. (**C**) Gating strategy for flow cytometry experiments. (i) Collected data was first gated on whole cells to exclude debris. (ii) Doublets of cells were excluded. (iii) Cells with a chromatin content ranging form 1n to 2n were included in the data. (iv) The resulting histogram for the PI intensities (FL-3 H) is shown. G1/G0 content was quantified by measuring the integral from half of the G1 peak starting on the left side to the peak of G1 as indicated and multiplication by 2. G2/M content was quantified similarly by starting the integral from the peak of G2 to the right side and multiplication by 2. The S content is quantified by subtraction of G1/G0 and G2/M content from 100% of the population. (**D**) Cells were treated with 5 ng/ml tox^{WT} or tox^{HQ} for 24h. 30,000 cells were typically analysed per sample. Overlay of representative cell cycle profiles to the right. (**E**) Cell-cycle arrest in toxin-treated cells. HT1080 cells either untreated (unt) or treated for 2h with toxin^{WT} or toxin^{HQ} were analysed at indicated times by flow cytometry and propidium iodide staining, which was used as DNA marker and fluorochrome. Data points indicate technical replicates (8 biological replicates, 80,000 cells per variable). Statistical significance (**** = P<0.0001, *** = P<0.0002, ** = P<0.0021, * = P< 0.0332, ns = P>=0.05) was assessed relative to untreated control using two-way ANOVA and a Dunnett's multiple comparison test. Error bars SD. Source data are provided as a Source Data file.

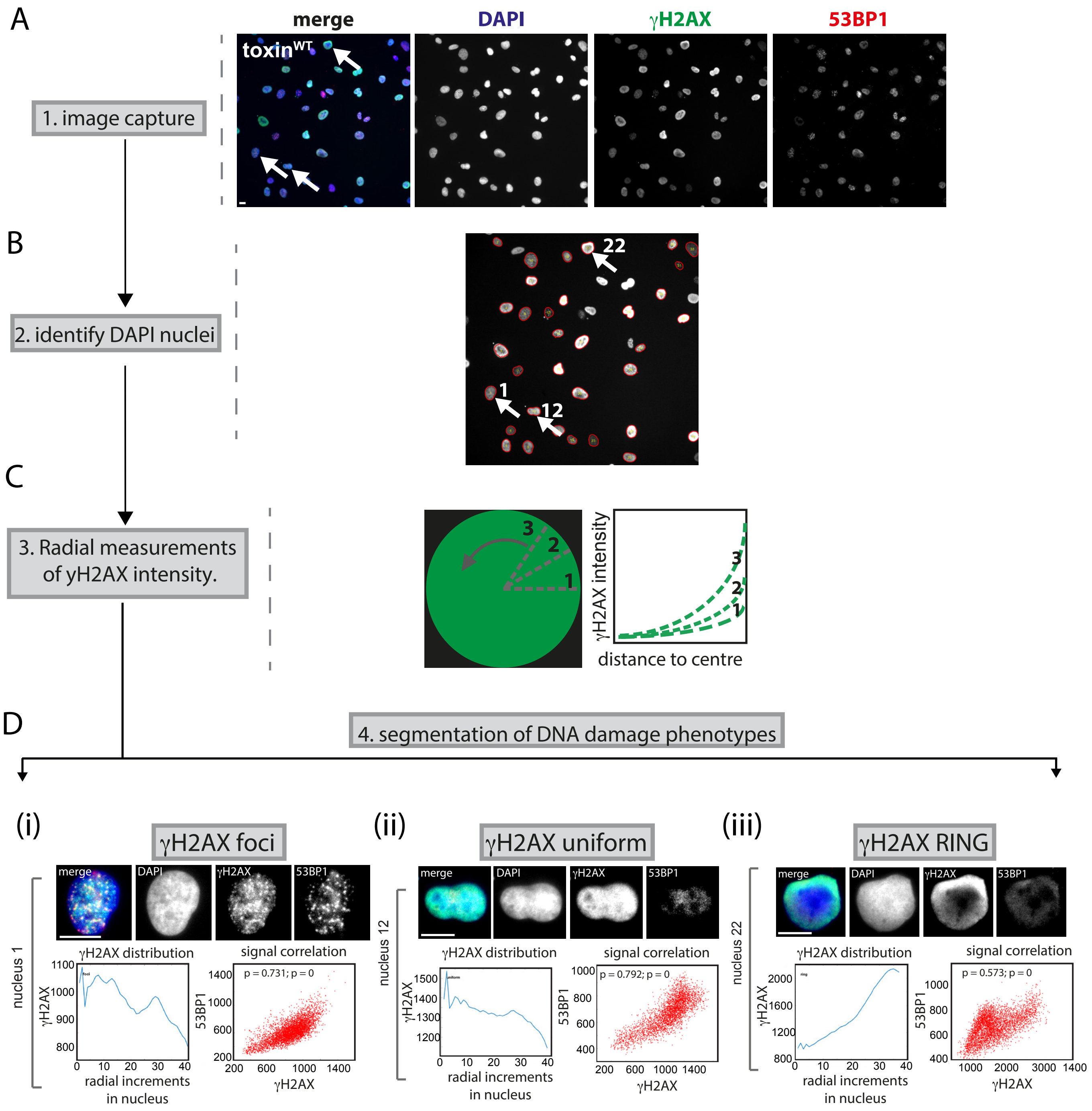

Supplementary Figure 2. Automated RING Tracking analysis

(**A**) Image capture. Mammalian cells intoxicated for 2h with toxin^{WT} were imaged at 24h following labelling with antibodies to γH2AX (green) and 53BP1 (red) and DAPI (blue). Images were analysed using a custom-made script in MATLAB as depicted in B-E and fully described in materials and methods. Arrows and numbers indicate example cells analysed in D. (**B**) Identify DAPI nuclei. All nuclei were detected in the DAPI channel. Arrows and numbers indicate example nuclei analysed in D. (**C**) Radial measurements of γH2AX intensity. Nuclei with the number of foci divided by the nucleus surface area being greater than 0.0035 were considered as having a γH2AX foci phenotype. As depicted in the cartoon, nuclei with non-foci γH2AX distribution were then further classified by measuring radial γH2AX intensities from the centre of each nucleus to the nuclear periphery. (**D**) Segmentation of DNA damage phenotypes into (i) γH2AX foci, (ii) γH2AX uniform or (iii) γH2AX RINGs. The nucleus was classified as a RING if the difference between mean γH2AX intensity in its last quartile and first three quartiles was greater than 10% of overall mean intensity. Fluorescent images indicate example phenotypes for i-iii with radial γH2AX intensity plots shown bottom left. The signal correlation between γH2AX and 53BP1 was determined by calculating the pairwise correlation coefficient between their intensities at each pixel within individual nuclei as indicated by the plots bottom right. Source data are provided as a Source Data file.

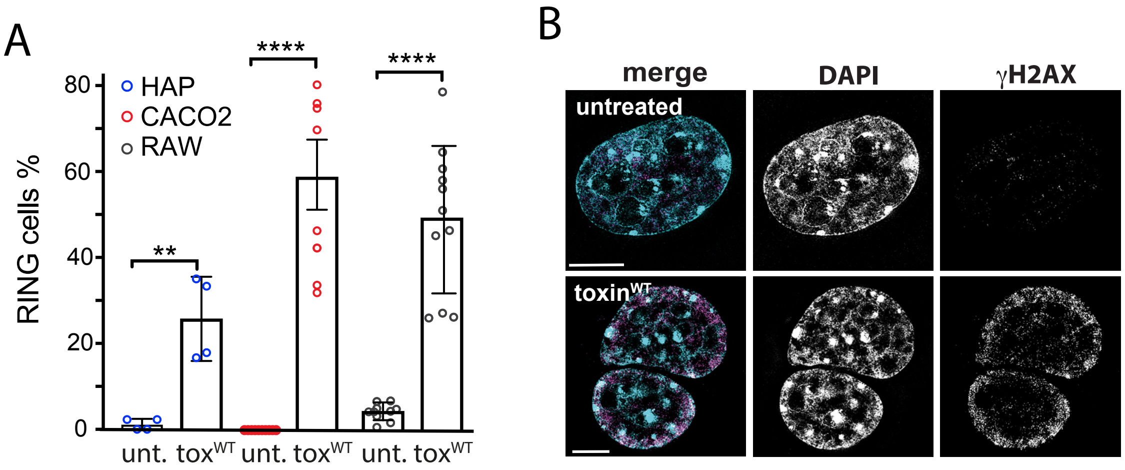

Supplementary Figure 3. Rings of γH2AX in distinct cell lines

(**A**) Proportion of RINGs in HAP, CACO2 and RAW267.4 cell lines at 24h following incubation with tox^{WT}. Untreated used as control. Blue circles represent means from 4 biological replicates and red/grey circles indicate means from fields of view in at least 2 biological replicates (at least 93 nuclei per variable analysed). Statistical significance (**** = P<0.0001, *** = P<0.0002, ** = P<0.0021, * = P< 0.0332, ns = P>=0.05) was calculated relative to untreated in each cell line using an unpaired two-sided *t* test. Error bars SD. (**B**) Representative structured illumination microscopy image of γH2AX RING (magenta) in the nucleus in MEF cells treated with the typhoid toxin. DAPI-stained nuclei shown (cyan). Scale bars 5μm. Source data are provided as a Source Data file.

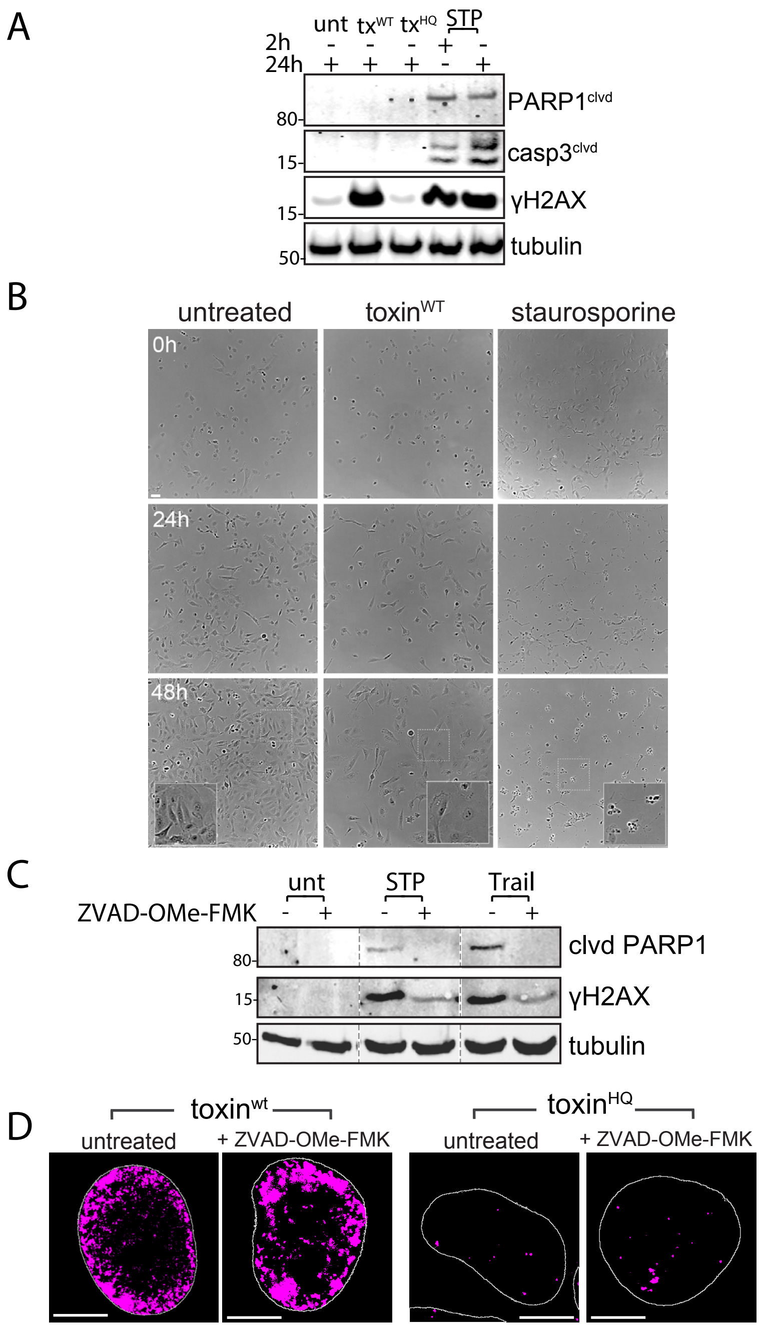

Supplementary Figure 4. Toxin-induced phenotypes are independent of apoptosis

(**A**) Apoptotic-signalling in cells treated with the typhoid toxin. Whole cell lysates derived from HT1080 cells either untreated (unt) or treated for 2h with recombinant wild-type toxin^{WT} (tx^{WT}) or toxin^{HQ} (tx^{HQ}) were analysed after 24h by immunoblotting with antibodies to cleaved PARP1 (PARP1^{clvd}), cleaved caspase3 (casp3^{clvd}), γH2AX or tubulin as a loading control. The positive control, staurosporine (STP), was added for 2h or 24h and used as an inducer of apoptosis. Molecular weight markers in kDa indicated left. (**B**) Still images captured from time-lapse microscopy of untreated cells, or cells treated with for 2h with either toxin^{WT} or staurosporine. Time-points indicated. Insets magnify confluent cells (untreated), distended cells (toxin^{WT}) or apoptotic bodies (staurosporine). Scale bar 50μm. (**C**) Apoptotic-signalling in the presence of an apoptosis inhibitor. HT1080 cells either untreated (unt) or treated with inducers of apoptosis staurosporine (STP) or Killer-Trail (Trail) in the presence (+) or absence (-) of the pan-caspase inhibitor ZVAD-OMe-FMK for 24h. Whole cell lysates were immunoblotted with antibodies to PARP1^{clvd}, γH2AX or tubulin as a loading control. Dashed lines indicate omitted extraneous portions of blot. Molecular weight markers in kDa indicated left. (**D**) RING formation in the presence of an apoptosis inhibitor. HT1080 cells either treated for 2h with recombinant wild-type toxin^{WT} or toxin^{HQ} in the presence (ZVAD-OMe-FMK) or absence (untreated) of a pan-caspase inhibitor were imaged at 24h following labelling with antibodies to γH2AX (magenta) and labelling of DAPI-stained nuclei (outlined). Scale bars 5μm. Source data are provided as a Source Data file.

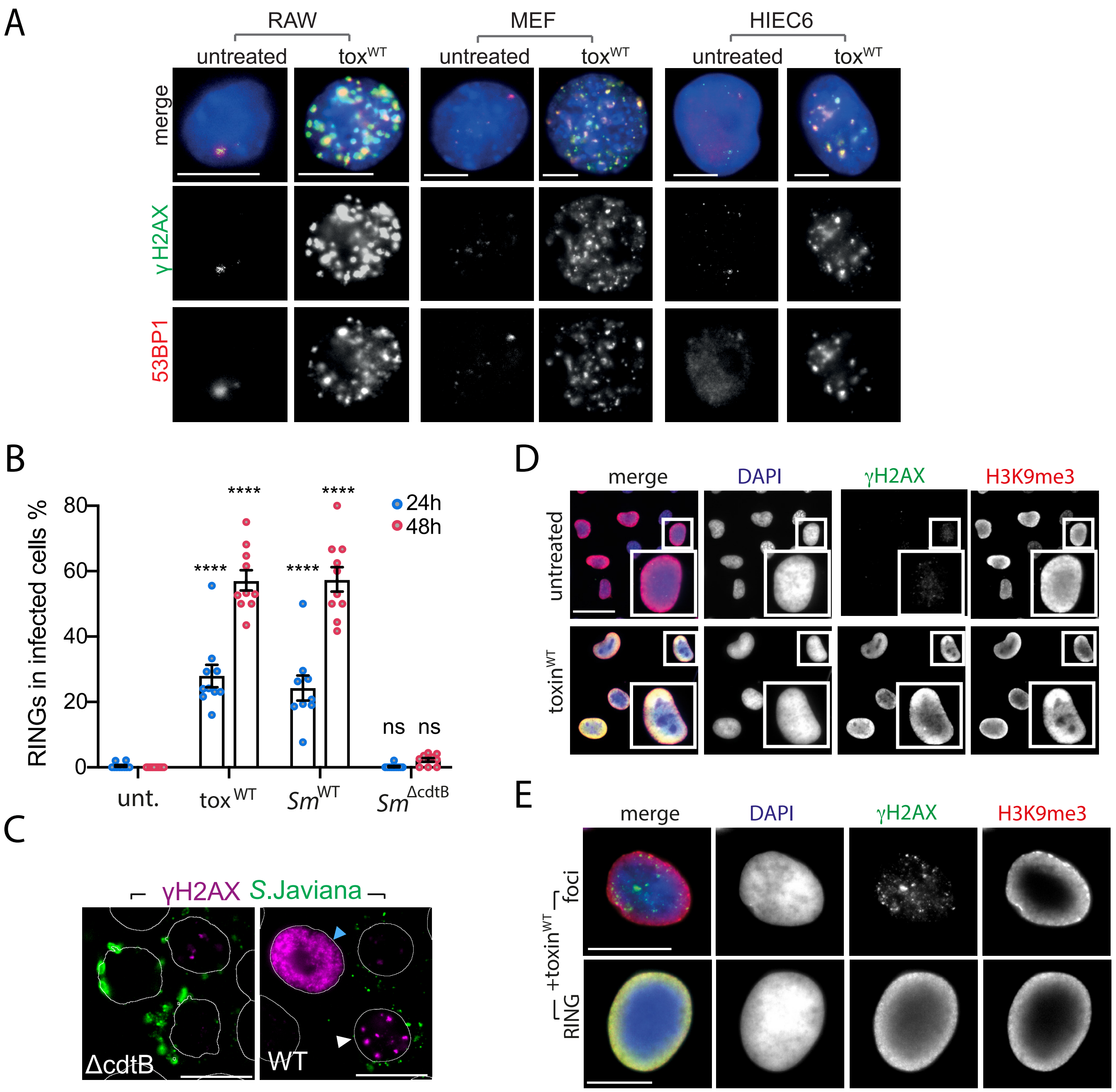

Supplementary Figure 5. Rings of γH2AX at heterochromatin and during infection

(**A**) Representative images of γH2AX (green) and 53BP1 (red) foci in untreated and toxin^{WT}-treated RAW macrophage, mouse embryonic fibroblasts (MEF) and human intestinal epithelial cells (HIEC6). DAPI-stained nuclei are shown (blue). Scale bars 5μm. (**B**) Proportion of cells with γH2AX RINGs at 24h or 48h following 1h infection with *S.*Typhi encoding typhoid toxin (*Sm*^{WT}) or toxin-deficient mutant (*Sm*$^{\Delta cdtB}$). Untreated and toxin^{WT} used as controls. At least 7 technical replicates (400 nuclei per variable). Error bars SEM. Statistical significance (**** = P<0.0001, *** = P<0.0002, ** = P<0.0021, * = P< 0.0332, ns = P>=0.05) was calculated relative to corresponding untreated control using two-way ANOVA and a Dunnett's multiple comparison test. (**C**) Representative image of γH2AX RING in cells infected with wild-type *S.*Javiana. Toxin-deficient mutant used as negative control (Δ*cdtB*). γH2AX labelled in magenta and outlines of DAPI-stained nuclei are shown. γH2AX RING (blue arrow) or foci (white arrow). Scale bars 20μm. (**D**) Representative images of of γH2AX (green) and heterochromatin marker H3K9me3 (red) in untreated and intoxicated (tox^{WT}) HT1080 cells. Insets magnify γH2AX and heterochromatin H3K9me3 localisation. DAPI-stained nuclei are shown (blue). Scale bars 20μm. (**E**) Localisation of heterochromatin marker H3K9me3 (red) in intoxicated cells with either a γH2AX (green) foci or RING phenotype. DAPI-stained nuclei are shown (blue). Scale bars 10μm. Source data are provided as a Source Data file.

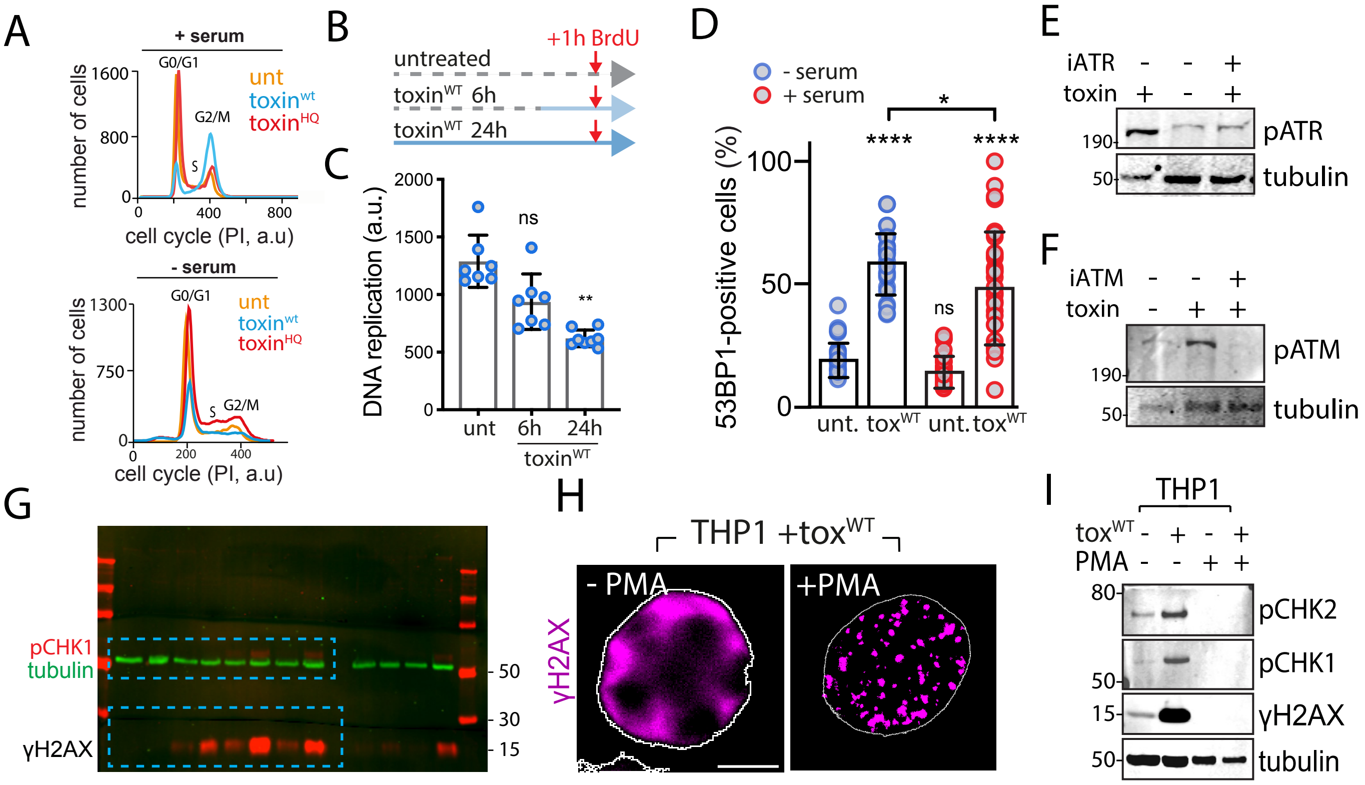

Supplementary Figure 6. RINGs require ATR

(**A**) Representative cell-cycle phases (G_0G_1, S, $G_{2/}M$) in intoxicated replicating (+ serum) and non-replicating (-serum) Ht1080 cells. Propidium iodide (PI) used as DNA marker and fluorochrome (fluorescence shown in arbitrary units, a.u). Top plot duplicated from Fig 1C for clarity. (**B**) Experimental outline for toxin-inhibition of DNA replication in (C). HT1080 cells were untreated or treated with with toxin^{WT} for 2h then incubated for either 6h or 24h as indicated. (**C**) Toxin-inhibition of DNA replication. Nucleotide analogue BrdU was added to cells 1h before fixation then proportion of cells with BrdU-labelled DNA quantified. Circles indicate means from fields of view in 2 biological replicates (2000 nuclei /variable). A.U, arbitrary units. (**D**) Proportion of 53BP1-positive cells following incubation with toxin^{WT} -/+serum. Untreated used as control. Coloured circles represent means of technical replicates (3 biological replicates, 530 nuclei per variable). (**E**) Immunoblot of pATR (anti-ATR pSer428) in intoxicated HT1080 cells -/+ 24h incubation with ATR inhibitor (iATR). Tubulin loading control. MW markers in KDa left. (**F**) Immunoblot of pATM (anti-ATM pSer1981) in intoxicated HT1080 cells -/+ 24h incubation with ATM inhibitor (iATM). Tubulin loading control. MW markers in KDa left. (**G**) Complete immunoblot used to generate Fig 2D. Crop indicated with blue box. MW in kDa right. (**H**) Replicating THP1 cells (-PMA) or non-replicating THP1s (+PMA) were treated with toxin^{WT} before assaying γH2AX RING formation using antibodies to γH2AX (magenta). Outlines of DAPI-stained nuclei are shown. Scale bars 5μm. (**I**) Immunoblotting of THP1 cells from (H) with indicated antibodies. MW in kDa left. Statistical significance (**** = P<0.0001, *** = P<0.0002, ** = P<0.0021, * = P< 0.0332, ns = P>=0.05) was calculated relative to control using one-way ANOVA together with a Dunnett's (**C**) or Tukey's (**D**) multiple comparison test. Error bars, SD. Source data are provided as a Source Data file.

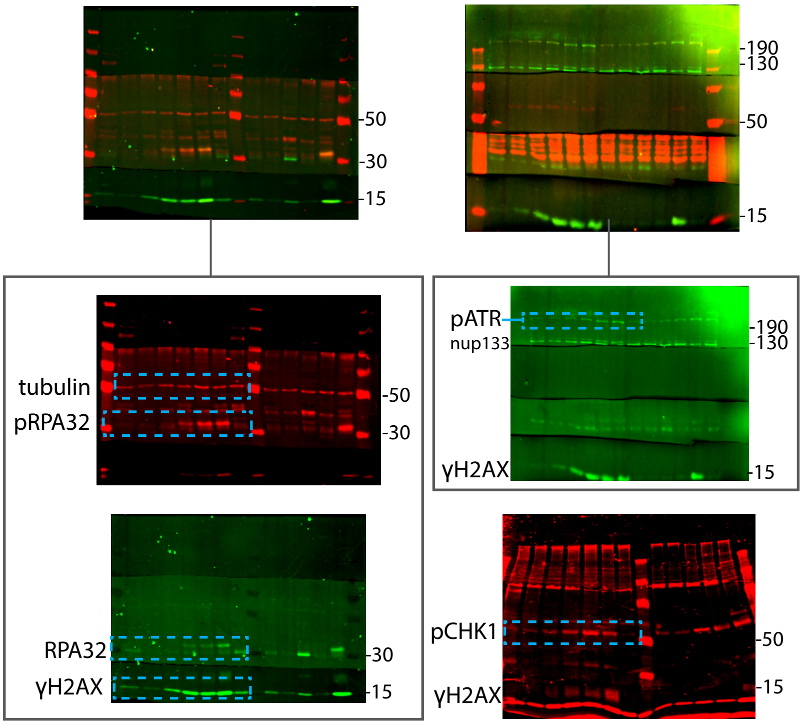

Supplementary Figure 7. Immunoblots relating to Fig 3A

Unprocessed immunoblots used to generate Fig 3A. Crops used for figure indicated with blue boxes. Antibodies indicated left. Blots in grey boxes shown in single colour channels for clarity. MW in kDa right. Source data are provided as a Source Data file.

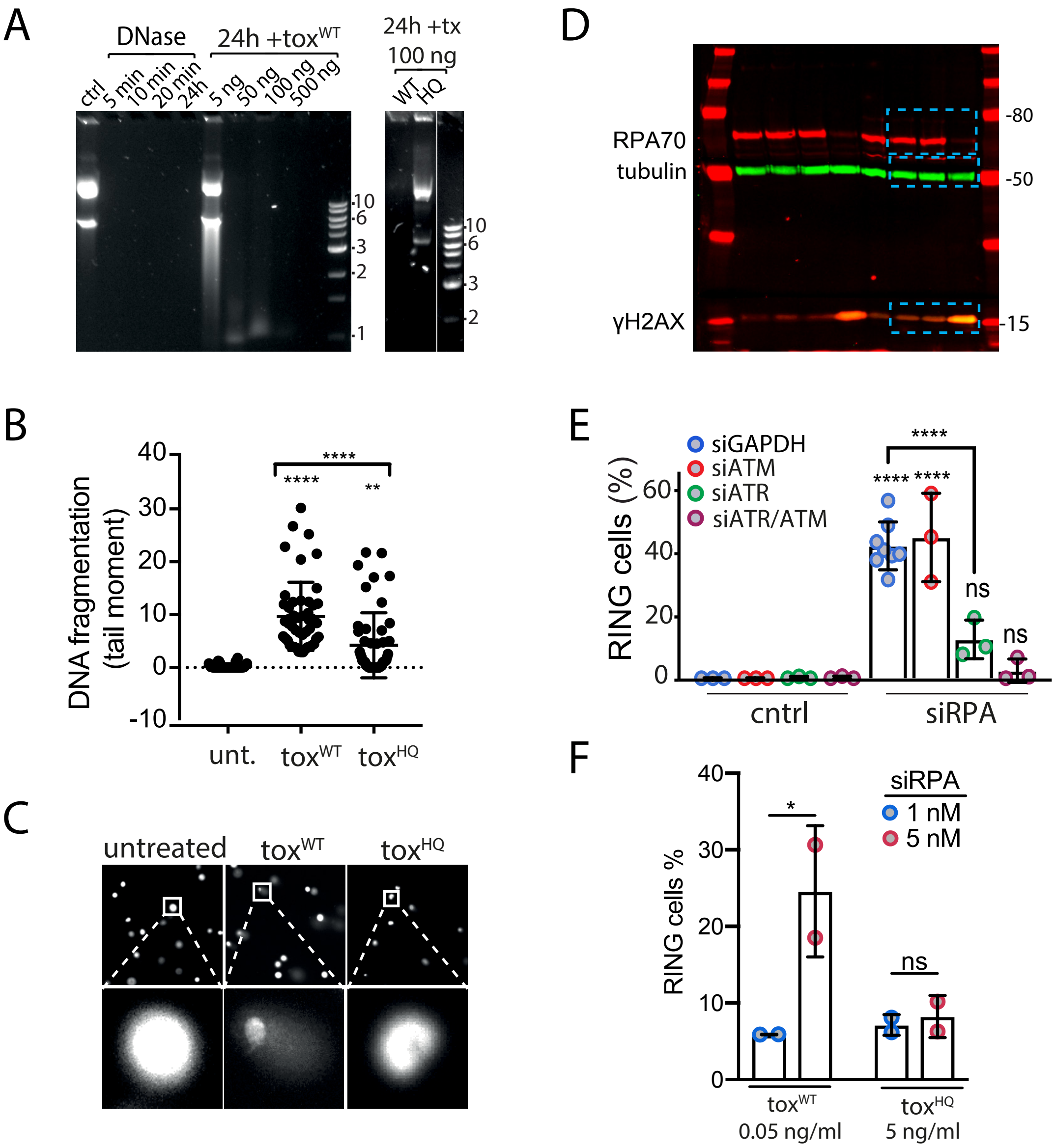

Supplementary Figure 8. Toxin breaks DNA and RPA exhaustion induces RINGs

(**A**) Representative *in vitro* DNA nuclease assay with typhoid toxin. pET-Duet1 plasmid DNA alone (ctrl) or in combination with 1Unit bovine DNase incubated for indicated timepoints was compared to plasmid DNA incubated with indicated amounts of toxin^{WT} or toxin^{HQ} for 24h. DNA markers Kb shown right. (**B**) Physical DNA strand breakage by the typhoid toxin. Bee plots showing alkaline comet assay analysis of DNA fragmentation (tail moment) in HT1080 cells either untreated, or treated with tox^{WT} or tox^{HQ}. Circles indicate individual cells in 3 biological replicates, 50 nuclei in total. (**C**) Representative images of fragmented DNA in toxin-treated cells from alkaline comet assays. Insets magnify cell DNA. (**D**) Unprocessed immunoblots used to generate Fig 3D. Crops used for figure indicated with blue boxes. Antibodies indicated left. MW in kDa right. (**E**) Proportion of γH2AX RINGs in RPA knockdown cells in presence of ATR or ATM siRNAs, or GAPDH siRNA as control. Circles indicate means from at least 3 biological replicates (>200 nuclei per variable). (**F**) Proportion of 53BP1-positive cells following incubation with toxin^{WT} -/+serum. Untreated used as control. Coloured circles represent means of 2 biological replicates (367 nuclei per variable). Statistical significance (**** = P<0.0001, *** = P<0.0002, ** = P<0.0021, * = P< 0.0332, ns = P>=0.05) was calculated relative to corresponding control using one-way ANOVA and either a Tukey's multiple comparison test (**B, E**), or two-way ANOVA and a Sidak's multiple comparison test (**F**). Error bars SD. Source data are provided as a Source Data file.

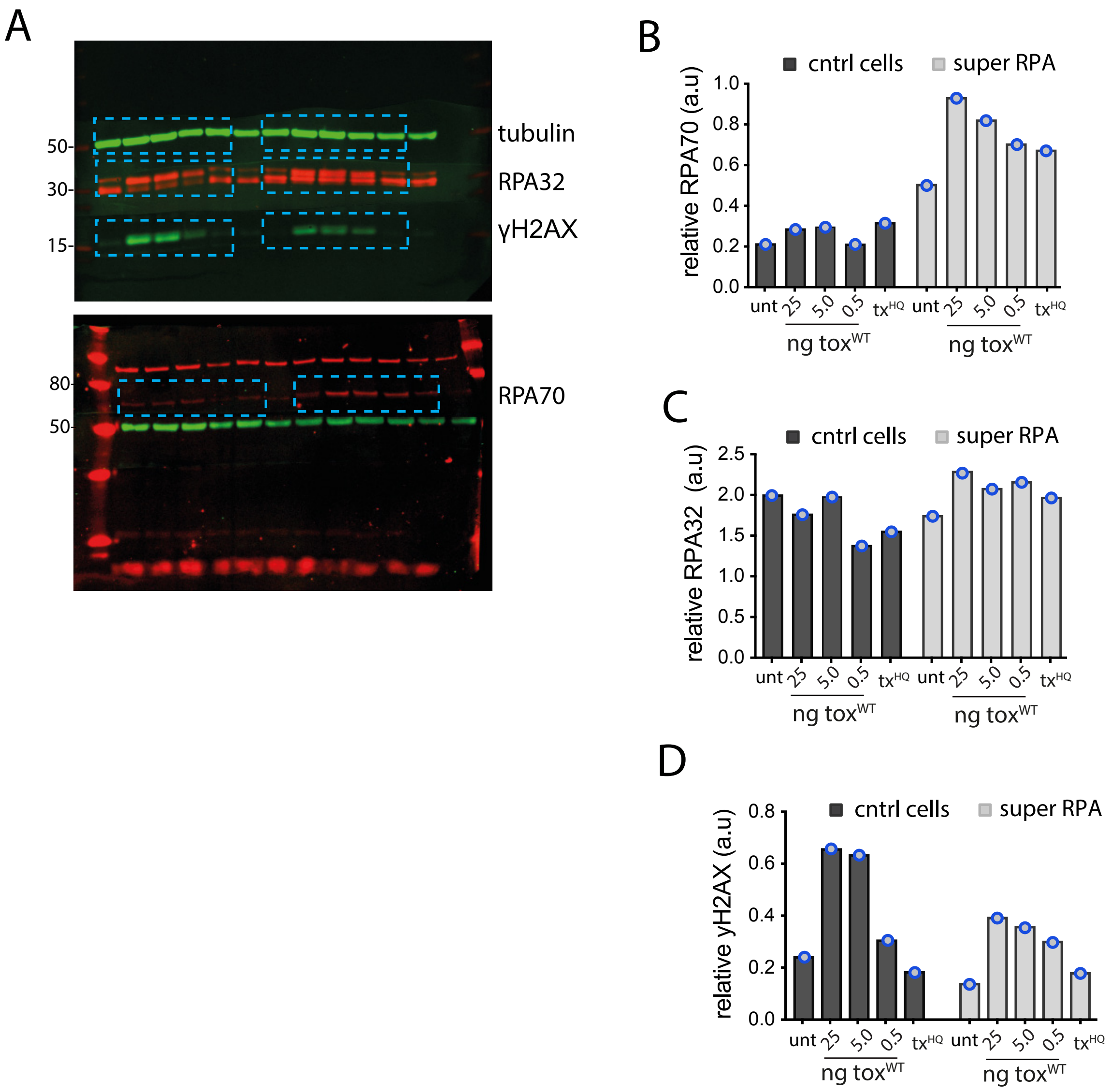

Supplementary Figure 9. Immunoblots relating to Fig 3H

(**A**) Unprocessed immunoblots used to generate Fig 3H. Crops used for figure indicated with blue boxes. Antibodies indicated right. MW in kDa left. Quantification of relative band intensities of (**B**) RPA70, (**C**) RPA32, and (**D**) γH2AX in Fig 3H assaying control and super RPA U2OS cells treated with indicated concentrations of tox^{WT} or 25ng/ml tox^{HQ}. Blue circles indicate relative band intensity values (1 biological replicate). Source data are provided as a Source Data file.

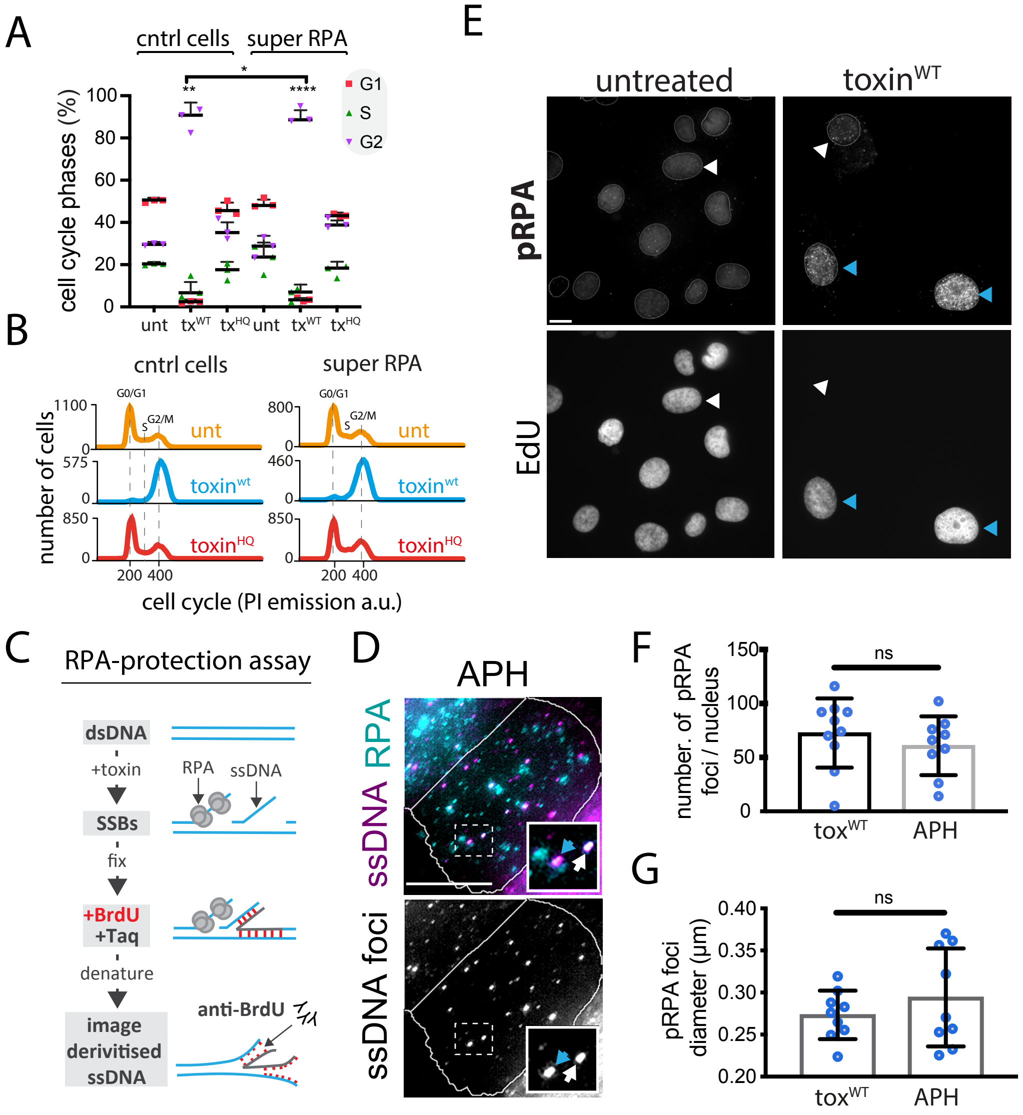

Supplementary Figure 10. Toxin exhaustion of RPA

(**A**) Cell-cycle arrest in control or super RPA U2OS cells treated with the typhoid toxin. Individual symbols indicate means from 3 biological replicates, ~30,000 cells per sample. Colours indicate cell-cycle phases as presented in key. (**B**) Representative images of toxin-induced cell-cycle arrest from (A). Cell-cycle phases indicated with dashed line. PI is propidium iodide used to label DNA and used as fluorochrome (fluorescence shown in arbitrary units, a.u). (**C**) Cartoon depicting RPA-protection assay. dsDNA is damaged by the toxin inducing single-strand DNA breaks (SSBs), which are coated by RPA to protect ssDNA. If left unprotected, ssDNA can be used to polymerise dsDNA using nucleotide analogue BrdU and Taq DNA polymerase, which is imaged to identify sites of unprotected ssDNA. (**D**) RPA Protection assay in the presence of aphidicolin (APH). HT1080 cells were treated for 24h with APH before polymerisation of double-stranded BrdU-labelled DNA from sites of exposed ssDNA template at 24h (ssDNA). ssDNA-bound RPA32 pT21 (RPA, cyan) with BrdU (magenta), also shown in grayscale for clarity (ssDNA foci). Outlines of DAPI-stained nuclei are shown. Insets magnify ssDNA foci unprotected by RPA (blue arrow) or partially protected by RPA indicated by white co-localisation staining (white arrow). Scale bars 10µm. (**E**) Representative images of EdU-labelled DNA (grayscale) and RPA32 pT21 (pRPA, grayscale) in toxin^{WT} treated cells at 24h. EdU incubated from 2h intoxication. Blue arrows indicate pRPA- and Edu-positive cells (toxin^{WT}). White arrows indicate EdU-positive cells with no pRPA foci (untreated) or EdU-negative cells with pRPA foci (toxin^{WT}). Outlines of DAPI-stained nuclei shown. Scale bars 10µm. (**F**) Number and (**G**) size of RPA32 pT21 (pRPA) foci in toxin^{WT}- or APH-treated cells. pRPA foci were quantified from the cells in experiment in Fig 3C (toxin^{WT}) or Fig 4E (APH). Circles indicate means from fields of view in 3 technical replicates, 500 nuclei per variable (1 biological replicate). Statistical significance (**** = P<0.0001, *** = P<0.0002, ** = P<0.0021, * = P< 0.0332, ns = P>=0.05) was calculated relative to untreated G1 phase using two-way ANOVA and a Tukey's multiple comparison test (**A**), or an unpaired two-sided *t* test (**F, G**). Error bars, SD. Source data are provided as a Source Data file.

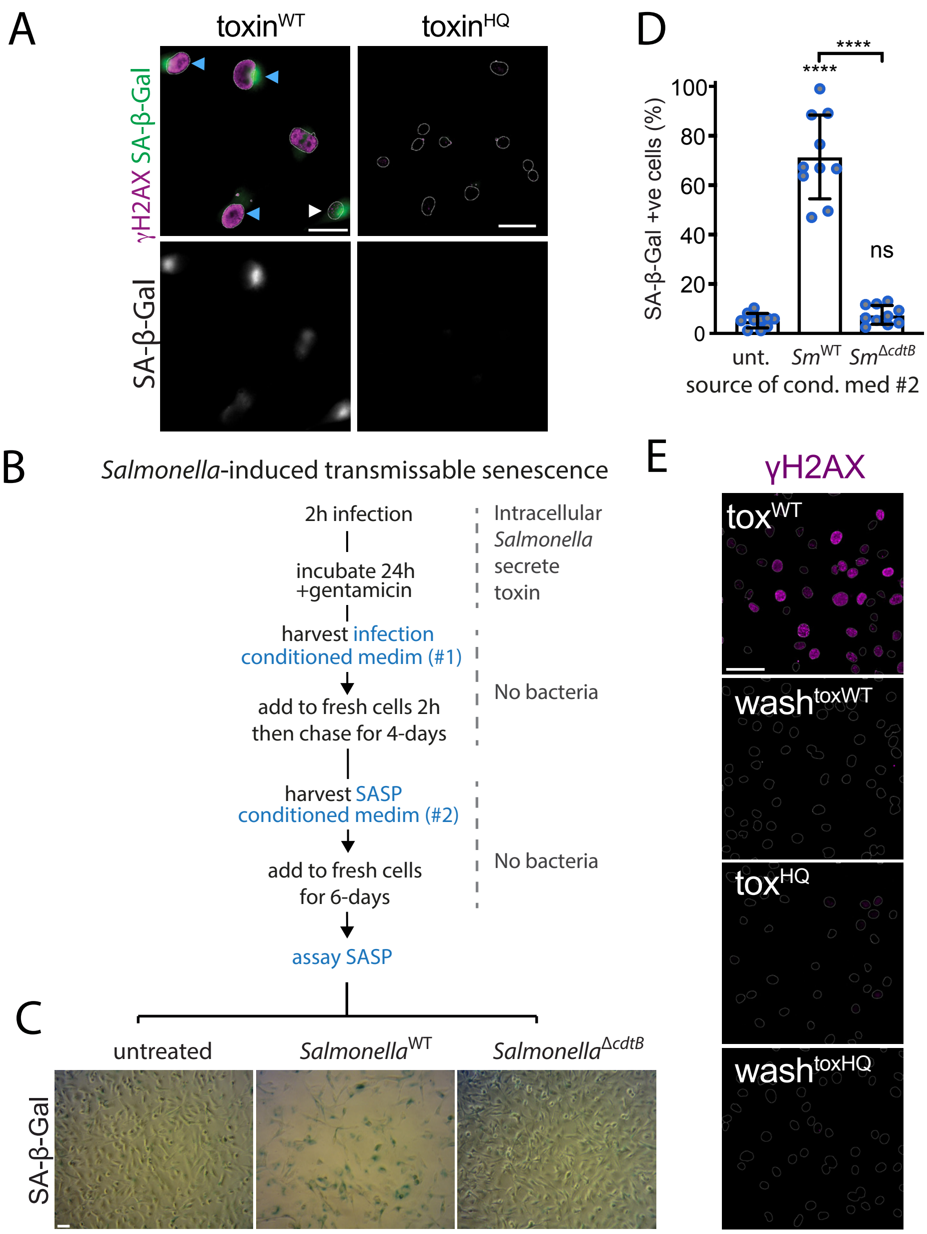

Supplementary Figure 11. Toxin induced senescence-like phenotype.

(**A**) Representative image of SA-β-Galactosidase activity at 48h following treatment with toxin^{WT} or toxin^{HQ}. Cells with SA-β-Galactosidase activity were visualised using SPiDER β-Gal reagent (green and grayscale) while γH2AX (magenta) was used to mark γH2AX RING cells (blue arrows) or cells with γH2AX foci (white arrow). Outlines of DAPI stained nuclei shown. Scale bars 50µm. (**B**) *Salmonella*-induced transmissible senescence experiment controlling for toxin secreted by intracellular *Salmonella* during infection (experimental workflow relating to Fig S8C, S8D). HT1080 cells were either untreated or infected with *Salmonella* Javiana (WT or Δ*cdtB*) before incubation with gentamicin for 24h to kill extracellular bacteria. During this 24h, intracellular *Salmonella* secrete the typhoid toxin into conditioned medium, which is harvested (infection conditioned medium #1). The conditioned medium #1 contains no bacteria and is used as a source of typhoid toxin to intoxicate fresh cells for 2h, which is removed prior to cell incubation in fresh media containing antibiotics. After 4-days, the conditioned medium containing secreted host proteins (SASP conditioned medium #2) is harvested and added to fresh cells for 4-days before assaying SASP by SA-β-Gal imaging in (**C**), scale bars 100µm. (**D**) Proportion of cells with SA-β-Gal from experiment (B). Statistical significance (**** = P<0.0001, ns = P>=0.05) was calculated relative to untreated control using one-way ANOVA and a Tukey's multiple comparison test. Error bars, SD. 2 technical replicates, 2000 nuclei/variable (1 biological replicate). (**E**) Influence of toxin wash fractions on DNA damage. Following incubation with toxin^{WT}, wash fractions were harvested and used to treat cells before assaying γH2AX (magenta). Scale bars 50µm. Relates to Fig 5I. Source data are provided as a Source Data file.

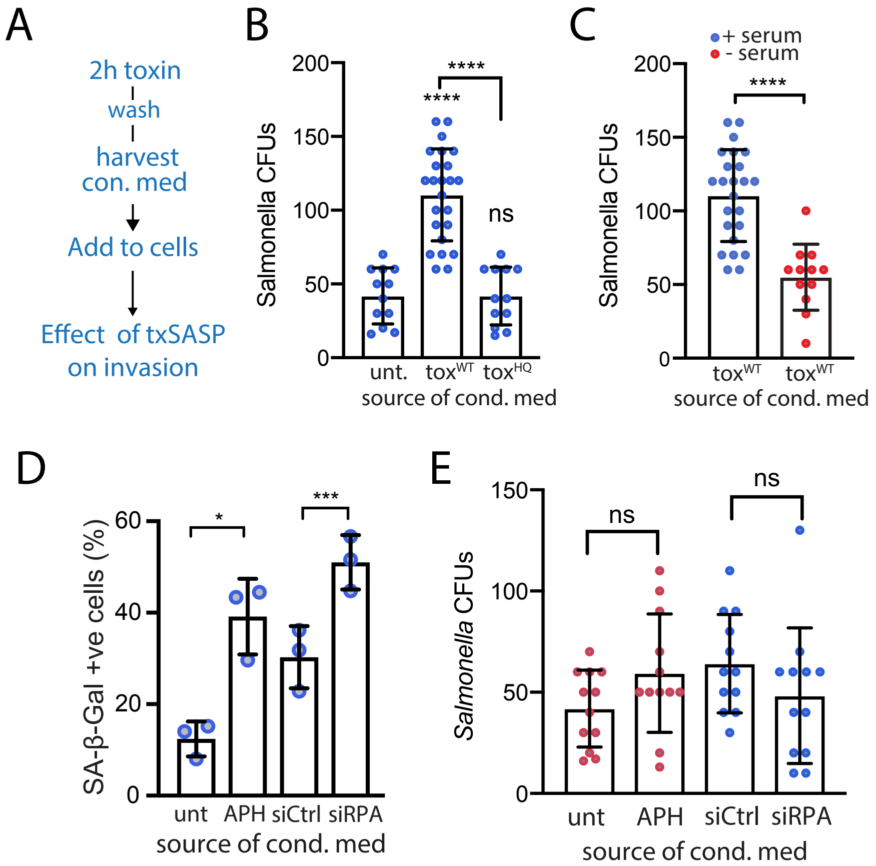

Supplementary Figure 12. Influence of txSASP on *Salmonella* invasion.

(**A**) Influence of txSASP on *Salmonella* invasion into HT1080 cells (experimental workflow). Conditioned media (con. med) was harvested from HT1080 cells after 6-days of treatment with toxin^{WT} or toxin^{HQ}, or no treatment as control. Conditioned media was incubated with fresh HT1080 cells before assaying the effect of txSASP on *Salmonella* invasion into cells. (**B**) TxSASP promotes *Salmonella* invasion into host cells. Serum-starved HT1080 cells incubated for 1h with conditioned medium from (A) before infection with *S*.Javiana (30min) and incubation with genatimicin 2h. CFUs calculated from colony counts. At least 6 technical replicates per variable (2 biological replicates). (**C**) TxSASP promotes *Salmonella* invasion in RING permissive conditions (+serum). Experiment performed as (B) using conditioned medium harvested -/+serum. CFUs calculated from colony counts in 6 technical replicates per variable (2 biological replicates). (**D**) Transmissible senescence by aphidicolin and RPA knockdown cells. HT1080 cells were incubated with aphidicolin (APH) for 24h or siRPA for 48h before harvesting conditioned medium at 4-days, which was used to treat fresh cells and assay transmissible senescence by quantifying the proportion of SA-β-Gal-positive cells. Circles indicate means from 3 biological replicates, ~2,600 cells analysed per variable (**E**) Influence of conditioned media from aphidicolin-treated (APH) and RPA-knockdown cells on *Salmonella* invasion. CFUs were calculated from colony counts in 6 technical replicates per variable (2 biological replicates). Statistical significance (**** = P<0.0001, *** = P<0.0002, ** = P<0.0021, * = P< 0.0332, ns = P>=0.05) was assessed relative to control using one-way ANOVA and a Tukey's multiple comparison test (**B, D**), or an unpaired two-sided *t* test (**C, E**). Error bars, SD. Source data are provided as a Source Data file.
