# Supplementary material for: Typhoid toxin exhausts the RPA response to DNA replication stress driving senescence and Salmonella infection
Source: Nat Commun. 2019 Sep 6;10:4040. doi: 10.1038/s41467-019-12064-1 (PMC6731267; doi:10.1038/s41467-019-12064-1)
Supplement: Supplementary file 3 — Reporting Summary [file 41467_2019_12064_MOESM3_ESM.pdf]

nature research

Corresponding author(s): Daniel Humphreys

Last updated by author(s): Aug 6, 2019

Reporting Summary

Nature Research wishes to improve the reproducibility of the work that we publish. This form provides structure for consistency and transparency in reporting. For further information on Nature Research policies, see Authors & Referees and the Editorial Policy Checklist.

Statistics

For all statistical analyses, confirm that the following items are present in the figure legend, table legend, main text, or Methods section.

n/a	Confirmed	
☐	☒	The exact sample size (n) for each experimental group/condition, given as a discrete number and unit of measurement
☐	☒	A statement on whether measurements were taken from distinct samples or whether the same sample was measured repeatedly
☐	☒	The statistical test(s) used AND whether they are one- or two-sided<br>*Only common tests should be described solely by name; describe more complex techniques in the Methods section.*
☒	☐	A description of all covariates tested
☒	☐	A description of any assumptions or corrections, such as tests of normality and adjustment for multiple comparisons
☐	☒	A full description of the statistical parameters including central tendency (e.g. means) or other basic estimates (e.g. regression coefficient) AND variation (e.g. standard deviation) or associated estimates of uncertainty (e.g. confidence intervals)
☐	☒	For null hypothesis testing, the test statistic (e.g. F, t, r) with confidence intervals, effect sizes, degrees of freedom and P value noted<br>*Give P values as exact values whenever suitable.*
☒	☐	For Bayesian analysis, information on the choice of priors and Markov chain Monte Carlo settings
☒	☐	For hierarchical and complex designs, identification of the appropriate level for tests and full reporting of outcomes
☒	☐	Estimates of effect sizes (e.g. Cohen's d, Pearson's r), indicating how they were calculated

Our web collection on statistics for biologists contains articles on many of the points above.

Software and code

Policy information about availability of computer code

Data collection	NIS-Elements AR software (Nikon, version 4.30.02), Zen (Zeiss, version 2.3SP1), MetaXpress (Molecular Devices, version 3.1), DeltaVision OMX softWoRx (GE Healthcare, version 6.0), Image Studio (LiCor, version 4.0), Image Lab (BioRad, version 5.0), DinoXcope Dino-Lite software (AnMo Electronics Corporation, version 1.19.2), CellQuest Pro (BD Biosciences, version 6.0)
Data analysis	GraphPad Prism 7 (GraphPad Software, version 7.01), ImageJ (National Institutes of Health, version 2.0.0-rc-54), FlowJo (FlowJo, version 10.4.1). Excel (Microsoft, version 16.16.4), MatLab (MathWorks, version R2018b), and Comet Assay IV (Instem, version IV)

For manuscripts utilizing custom algorithms or software that are central to the research but not yet described in published literature, software must be made available to editors/reviewers. We strongly encourage code deposition in a community repository (e.g. GitHub). See the Nature Research guidelines for submitting code & software for further information.

Data

Policy information about availability of data

All manuscripts must include a data availability statement. This statement should provide the following information, where applicable:

- Accession codes, unique identifiers, or web links for publicly available datasets
- A list of figures that have associated raw data
- A description of any restrictions on data availability

A data availability statement is provided and contains (i) weblink to publicly available datasets on figshare, (ii) a list of figures that have associated raw data in the source data file, and (iii) a statement on data availability, i.e. The authors declare that data supporting the findings of this study are available within the paper and its supplementary information files.

Field-specific reporting

Please select the one below that is the best fit for your research. If you are not sure, read the appropriate sections before making your selection.

☒ Life sciences ☐ Behavioural & social sciences ☐ Ecological, evolutionary & environmental sciences

For a reference copy of the document with all sections, see nature.com/documents/nr-reporting-summary-flat.pdf

Life sciences study design

All studies must disclose on these points even when the disclosure is negative.

Sample size	Sample size was chosen depending on the technique used and based on our experience (Methods: Data and Statistical Analysis).
Data exclusions	No data were excluded for experiments.
Replication	Experiments were replicated. The number of technical and biological replicates are indicated in each figure legend.
Randomization	Not relevant for the study, samples were not random.
Blinding	Investigators were blinded to the samples during data collection (i.e. fluorescence microscopy, Senescence β-Galactosidase Staining) and data analysis (i.e. Senescence β-Galactosidase Staining). Fluorescence microscopy data was typically analysed blind using RING Tracking (MatLab software) as indicated in the source data file.

Reporting for specific materials, systems and methods

We require information from authors about some types of materials, experimental systems and methods used in many studies. Here, indicate whether each material, system or method listed is relevant to your study. If you are not sure if a list item applies to your research, read the appropriate section before selecting a response.

Materials & experimental systems

n/a	Involved in the study	
☐	☒	Antibodies
☐	☒	Eukaryotic cell lines
☒	☐	Palaeontology
☒	☐	Animals and other organisms
☒	☐	Human research participants
☒	☐	Clinical data

Methods

n/a	Involved in the study	
☒	☐	ChIP-seq
☐	☒	Flow cytometry
☒	☐	MRI-based neuroimaging

Antibodies

Antibodies used	Antibodies were purchased from Abcam (Salmonella, ab35156; RPA70, ab79398; RPA32/RPA2, ab16850; RPA32pT21/pRPA, ab61065; tubulin, ab7291/ab52866; H3K9me3, ab176916), Cell Signalling Technology (CHK1-pSer345, 2341; CHK2-pThr68, 2197; ATR-pSer428, 2853; ATM pSer1981, 5883;cleaved PARP1, 5625; cleaved caspase-3, 9664; γH2AX, 9718), Bethyl laboratories (RPA32, A300-244A; RPA32-pS33/pRPA32, A300-246A), Sigma (FLAG M2, F3165), GenScript (Myc, A00172-200), Qiagen (His, 34660), Millipore (γH2AX, 05-636-I), Novusbio (53BP1, NB100-304), BD Bioscience (BrdU, 347580). Secondary antibodies were purchased from ThermoFisher Scientific (Alexa 488 donkey anti-mouse IgG, A-21202; Alexa 594 donkey anti-rabbit IgG, A-21207) andLiCor Biosciences (IRDye® 800CW Donkey anti-Mouse IgG, 925-32212; IRDye® 680RD Donkey anti-Rabbit IgG, 926-68073).
Validation	All antibodies used in the study are commercially available, validated according to manufacturers' manuals and largely by the literature. For example, RPA antibodies were chosen due to previous validation (Toledo et al, 2013).

Eukaryotic cell lines

Policy information about cell lines

Cell line source(s)	Source ATCC: HT1080 (human epithelial, male) #ATCC® CCL-121, THP1 (human monocyte, male) #ATCC® TIB-202™, Mouse embryonic fibroblast (male/female) #ATCC® SCRC-1008™, RAW264.7 (mouse macrophage, male) #ATCC® TIB-71™, U-2-OS (epithelial, female, ATCC identifier HTB-96) from Luis Toledo (Toledo et al 2013), HAP1 (human fibroblast-like) from Horizon Discovery Group, CACO2 (human male colon epithelial cells) #ATCC® HTB-37 and HIEC-6 (human intestinal epithelial cells) #ATCC® CRL-3266.
Authentication	Purchased from ATCC unless stated, in which case, cell lines were not authenticated.

Mycoplasma contamination	Tested and not contaminated
Commonly misidentified lines (See ICLAC register)	No commonly misidentified lines were used in this study.

Flow Cytometry

Plots

Confirm that:

☒ The axis labels state the marker and fluorochrome used (e.g. CD4-FITC).

☒ The axis scales are clearly visible. Include numbers along axes only for bottom left plot of group (a 'group' is an analysis of identical markers).

☒ All plots are contour plots with outliers or pseudocolor plots.

☒ A numerical value for number of cells or percentage (with statistics) is provided.

Methodology

Sample preparation	Cells were seeded into 6-well plates (9.5 cm2/well) at a density of 30% confluency. HT1080 seeded at 3x105 cells per well. The next day, cells were treated with appropriate drugs or toxins as described in methods. After indicated incubation and chase time, cells were collected using a cell scraper and harvested by centrifugation (5min at 1200rpm, Rotina 46R , Hettich Zentrifugen) before resuspension in 200 µl PBS. Next, cells were fixed with 1 ml ice-cold 70% ethanol in PBS, briefly vortexed and put on ice for at least 30 min. For propidium iodide (PI) staining, cells were harvested (5min 4000rpm, Heraeus Pico17, Thermo Scientific) then resuspended in 300 µl flow cytometry buffer (PBS, 100 µg/ml RNase, 40 µg/ml PI) and incubated at 37C for 30 min to digest RNA which could impede the analysis of DNA content. Samples were stored on ice and analysed.
Instrument	FACSCalibur flow cytometer (Becton Dickinson) excitation wave length of 488 nm, FL1 was recorded at 530 nm emission, and FL3 was recorded at 650 nm emission.
Software	Data collection was performed with CellQuest Pro Version 6.0 software.<br>Data analysis was performed with FlowJo Version 10.4.1 software.
Cell population abundance	For serum-starved cells, 30,000 cells were collected on the instrument and 10,000 to 24,000 cells were included in the analysed fraction for each biological replicate.<br>For asynchronous cells, 10,000 to 40,000 cells were collected on the instrument and 5,000 to 30,000 cells were included in the analysed fraction for each biological replicate.
Gating strategy	Cells were gated for data collection using forward and sideward scatter, while 650 nm was recorded for PI emission of cells, and auto-fluorescence of cells was recorded at 530 nm. Collected data was gated for the right cell size with SSC and FSC, then doublets of cells were excluded by gating for amplitude of PI signal against the PI signal width, next gating for live cells was performed by including cells between G1 and G2 from graphs with FL3-H against FL1-H. Finally, histograms of FL3-H were generated and presented in the manuscript.<br>G1 population was measured as the integral from the lowest PI value of the G1 curve<br>to the peak of G1, and was multiplied by 2. The G2 population was measured as the integral from the highest PI value of the G2 curve to the peak of G2, and was multiplied by 2.

☒ Tick this box to confirm that a figure exemplifying the gating strategy is provided in the Supplementary Information.
